# Biannual versus annual mass azithromycin distribution and malaria seroepidemiology among preschool children in Niger: a sub-study of a cluster randomized trial

**DOI:** 10.1186/s12936-019-3033-2

**Published:** 2019-12-03

**Authors:** Catherine E. Oldenburg, Abdou Amza, Gretchen Cooley, Boubacar Kadri, Beido Nassirou, Benjamin F. Arnold, Philip J. Rosenthal, Kieran S. O’Brien, Sheila K. West, Robin L. Bailey, Travis C. Porco, Jeremy D. Keenan, Thomas M. Lietman, Diana L. Martin

**Affiliations:** 1 Francis I Proctor Foundation, University of California, San Francisco, San Francisco, CA USA; 2 Department of Ophthalmology, University of California, San Francisco, 513 Parnassus Ave, Room S334, San Francisco, CA 94143 USA; 3 Department of Epidemiology and Biostatistics, University of California, San Francisco, San Francisco, CA USA; 4 Programme FSS/Université Abdou Moumouni de Niamey, Programme Nationale des Soins Oculaire, Niamey, Niger; 5 Division of Parasitic Diseases and Malaria, Centers for Disease Prevention and Control, Atlanta, GA USA; 6 Department of Epidemiology, University of California, Berkeley, Berkeley, CA USA; 7 Department of Medicine, University of California, San Francisco, San Francisco, CA USA; 8 Dana Center for Preventive Ophthalmology, Wilmer Eye Institute, Johns Hopkins University, Baltimore, MD USA; 9 Clinical Research Unit, Department of Infectious and Tropical Diseases, London School of Hygiene and Tropical Medicine, London, UK

**Keywords:** Malaria, Azithromycin, Niger, Mass drug administration

## Abstract

**Background:**

Biannual mass azithromycin administration to preschool children reduces all-cause mortality, but the mechanism for the effect is not understood. Azithromycin has activity against malaria parasites, and malaria is a leading cause of child mortality in the Sahel. The effect of biannual versus annual azithromycin distribution for trachoma control on serological response to merozoite surface protein 1 (MSP-1_19_), a surrogate for malaria incidence, was evaluated among children in Niger.

**Methods:**

Markers of malaria exposure were measured in two arms of a factorial randomized controlled trial designed to evaluate targeted biannual azithromycin distribution to children under 12 years of age compared to annual azithromycin to the entire community for trachoma control (N = 12 communities per arm). Communities were treated for 36 months (6 versus 3 distributions). Dried blood spots were collected at 36 months among children ages 1–5 years, and MSP-1_19_ antibody levels were assessed using a bead-based multiplex assay to measure malaria seroprevalence.

**Results:**

Antibody results were available for 991 children. MSP-1_19_ seropositivity was 62.7% in the biannual distribution arm compared to 68.7% in the annual arm (prevalence ratio 0.91, 95% CI 0.83 to 1.00). Mean semi-quantitative antibody levels were lower in the biannual distribution arm compared to the annual arm (mean difference − 0.39, 95% CI − 0.05 to − 0.72).

**Conclusions:**

Targeted biannual azithromycin distribution was associated with lower malaria seroprevalence compared to that in a population that received annual distribution.

*Trial Registration* Clinicaltrials.gov NCT00792922

## Background

Biannual mass azithromycin administration has been shown to decrease all-cause post-neonatal child mortality in some settings in sub-Saharan Africa [1, 2]. Annual mass azithromycin distribution to entire communities is highly effective at clearing the ocular strains of chlamydia that cause blinding trachoma [3–5]. Since 1999, over 750 million doses of azithromycin have been distributed to trachoma-endemic districts [6, 7]. Mass drug administration (MDA) with azithromycin as part of trachoma elimination programmes has been shown to reduce the burden of other childhood infections, including diarrhoea [8], lower respiratory infection [9], and in some studies malaria [10, 11], possibly contributing to observed reductions in child mortality [12, 13].

Azithromycin has modest activity against *Plasmodium falciparum*, the most virulent human malaria parasite, due to action against the parasite apicoplast [14–17]. Previous studies assessing the effect of azithromycin MDA on malaria infection yielded contradictory conclusions. In Tanzania, malaria prevalence decreased transiently in the month following azithromycin MDA [11]. Previously, in the Partnership for the Rapid Elimination of Trachoma (PRET)-Niger study, lower malaria parasitaemia prevalence was documented in communities that had received two biannual azithromycin MDAs compared to communities receiving one annual MDA [10]. However, there was no difference in parasitaemia after 36 months of biannual versus annual MDA [18, 19]. These studies measured malaria infection via thick smear, 6 to 12 months after the last antibiotic dose, when trachoma measurements are typically performed in trachoma trials. The effect of a single dose of azithromycin for malaria would likely occur over a shorter time period, and thus a single measure of malaria infection many months after treatment may not be an ideal measure of the overall effect of azithromycin on malaria incidence. Thick smear will also not detect low-intensity infections [20]. Alternative measures of malaria exposure and transmission may therefore be useful in evaluating the effect of azithromycin for malaria as an off-target effect of azithromycin for trachoma control.

Antibody measurements have been proposed as markers of malaria exposure over time [21–23]. Population seroprevalence to merozoite surface protein 1 (MSP-1), a dominant antigen of asexual *P. falciparum*, is predicted to provide information about malaria incidence beyond point prevalence by integrating serologic responses at different time points [21–25]. While serologic markers are not direct measures of clinical burden, they provide seroprevalence and age-seroprevalence curves give insights into exposure and transmission patterns [22, 25]. Here, the effect of biannual distribution of azithromycin to children was compared to annual distribution to the entire community, each over three years, on the burden of malaria using serologic markers of *P. falciparum* exposure in preschool-aged children in Niger.

## Methods

### Ethical approval

Ethical approval was obtained from the Committee on Human Research at the University of California, San Francisco and the Comité d’Ethique du Niger. Verbal informed consent was obtained from local chiefs of each study community and from the parent or guardian of each study participant. CDC staff did not have contact with study personnel or access to personal identifying information and were determined to not be engaged in human subjects research.

### Study design

PRET was a series of community-randomized trials in Niger, The Gambia, and Tanzania designed to assess mass azithromycin distribution strategies for trachoma control (clinicaltrials.gov NCT00792922). In the present report, data from the Niger trial only were included [26–28]. The Niger trial was a 2 × 2 factorial trial of standard versus enhanced coverage and annual versus biannual distribution of azithromycin for trachoma control. In Niger, communities were randomized to one of four arms in a 1:1:1:1 fashion: (1) annual treatment of all individuals in the community with a treatment coverage target of 80%; (2) annual treatment of all individuals in the community with an enhanced treatment coverage target of 90%; (3) biannual treatment of children aged 12 and under with a treatment coverage target of 80%; or (4) biannual treatment of children aged 12 and under with an enhanced treatment coverage target of 90%. Communities were randomized by stratified block randomization within each Centre de Santé Intégrée (CSI) by high or low trachoma prevalence, as previously described [26]. The present report is restricted only to the enhanced coverage arms, as dried blood spots for antibody tested were only collected in these arms. The remainder of this report is, therefore, focused only on the enhanced distribution study arms. Communities were eligible for inclusion in the study if they had a population between 250 and 600 at the most recent government census (done in 2001 with population sizes in 2010 estimated based on projected population growth) and clinical trachoma prevalence of at least 10% at the time of the census.

### Study setting

Study communities were located in Matamèye District, Zinder Region and were treated from May 2010 until May 2013. This region is situated in the Sahel and has highly seasonal malaria incidence, with peak transmission shortly after the peak in rainfall, typically in September [29, 30]. At the time of the study, there was no seasonal malaria chemoprevention programme in this region, although a bed net distribution programme was active. Annual distributions occurred in June/July, at the beginning of the high transmission season. In the biannual distribution arm, communities were additionally treated in December/January, during the low transmission season. Data for the present analysis was collected in September 2013.

### Intervention

Prior to each MDA, a door-to-door enumerative census was undertaken in all study communities, which formed the sampling frame for treatment and evaluation. In all communities included in this report, each MDA occurred over a 1-to-4-day period: up to three follow-up visits occurred after the initial MDA day in an attempt to achieve coverage of 90% or greater. In the annual MDA arm, communities received a total of three rounds of MDA distributed via a door-to-door program to all individuals, regardless of age. In the biannual MDA communities, children aged 6 months to 12 years received a total of six rounds of door-to-door MDA; no one over the age of 12 was treated in these communities. During each MDA, all eligible participants were offered a single dose of directly observed oral azithromycin (20 mg/kg up to a maximum dose of 1 g in adults). Children under 6 months of age or those with macrolide allergy were offered topical tetracycline ointment (1%) for 6 weeks. In the annual treatment arm, pregnant women were offered tetracycline ointment.

### Serology assessment

In each study community, a random sample of 50 children aged 0 to 5 years were selected to have samples collected. Dried blood spots were only collected in children age 1 to 5 years of age due to the presence of maternal antibodies. Blood samples were collected via finger or heel stick in September 2013. Dried blood spots based on the most recent census [31] were analysed for antibody response to a portion of the *P. falciparum* antigen MSP-1_19_ using a multiplex bead array assay on a Luminex 200 platform. Results were reported as the median fluorescence intensity (MFI) minus background (MFI-BG), where background is the signal from beads with buffer only. The seropositivity cutoff was MFI-BG ≥ 1758 as determined by receiver operator characteristic curve analysis using a positive panel of serum from individuals with malaria slides positive for *P. falciparum* and a negative panel of serum from US-residents who had never travelled to a malaria-endemic region.

### Clinical and laboratory assessments

Blood samples from the children who contributed dried blood spots were also tested for *P. falciparum* infection using microscopy. Thick blood smears were stained with 3% Giemsa, and each slide was read by two experienced microscopists at the Zinder Regional Hospital. Discrepancies were adjudicated by a third reader. Microscopists determined the presence or absence of *P. falciparum* parasites and counted the number of asexual parasites per 200 white blood cells (assuming a white blood cell count of 8000/μl). Each child’s tympanic temperature was assessed at the time of blood collection. Clinically symptomatic malaria was defined as a blood slide positive for *P. falciparum* accompanied by tympanic temperature ≥ 38.5 °C. The geometric mean of the two parasite densities was used for analysis. Haemoglobin concentration was measured for all children (HemoCue AB, Ängelholm, Sweden).

### Sample size

The trial was powered for the primary trachoma outcome [26]. An a priori sample size calculation was not performed for this non-prespecified secondary outcome.

### Statistical methods

All analyses were conducted as intention-to-treat. Descriptive statistics were calculated by study arm with medians and interquartile ranges (IQR) or proportions. A log_10_ transformations of MFI-BG (as a semi-quantitative indicator of antibody levels) and parasite density was used for analysis. Parasite prevalence, clinically symptomatic malaria prevalence, and seroprevalence of MSP-1_19_-specific antibodies and corresponding binomial 95% confidence intervals (CI) were calculated at the community level. Prevalence ratios (PR) for the association between malaria infection and MSP-1_19_ seropositivity were calculated using generalized linear mixed models with a binomial distribution and log link with a random effect for the study community to account for clustering within communities, and adjusted for age and gender [32, 33].

To assess the difference in seropositivity to MSP-1 by study arm, generalized linear mixed model with a binomial distribution and log link were used to estimate risk ratios, with a random effect for study community. To assess differences in quantitative antibody levels, a log_10_ transformation of the MSP-1 MFI-BG values was used. Generalized linear mixed models with a Gaussian distribution and identity link were used to estimate the mean difference in antibody level between study arms, with a random effect for study community. Differences in the age-seroprevalence and semi-quantitative antibody curves were evaluated to assess differences in short- and long-term malaria exposure in a generalized linear model with binomial (seropositivity) or Gaussian (MFI-BG) distribution, with a study arm by age category interaction term, with age treated as a continuous variable. All analyses were conducted in R (version 3.4.3, The R Foundation for Statistical Computing, Vienna, Austria).

## Results

**Fig. 1:**
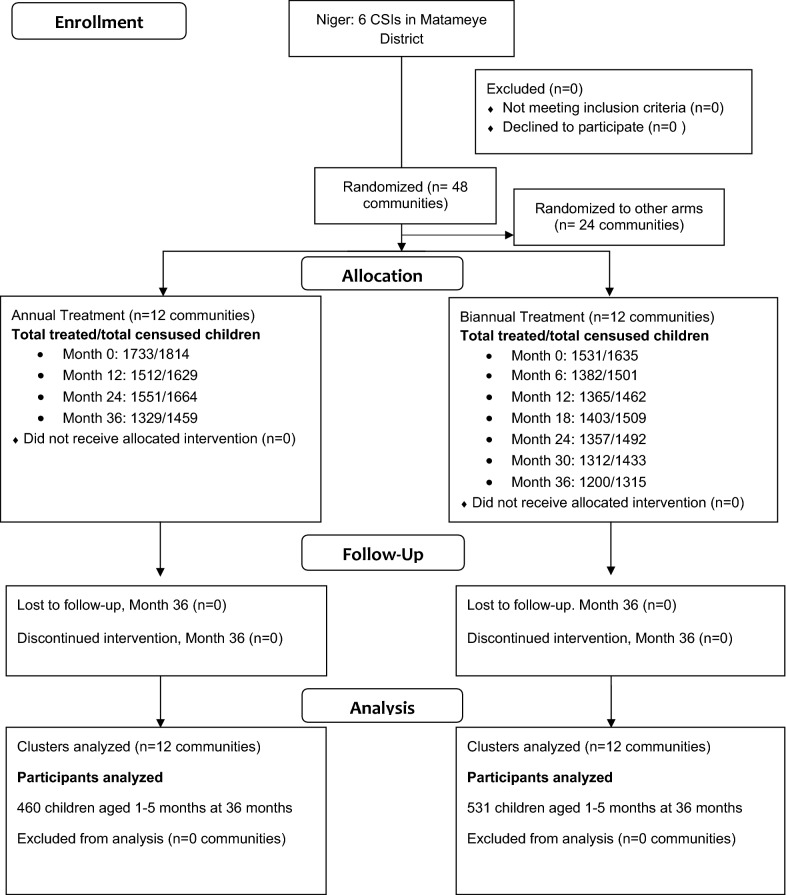
Consolidated Standards of Reporting Trials (CONSORT) diagram for included communities and individuals

A total of 991 children contributed MSP-1_19_ serologic data, of whom 460 were in annual azithromycin distribution communities and 531 in biannual azithromycin distribution communities (Fig. 1). The distribution of participant and baseline community characteristics was balanced between study arms (Table 1). Median age of children selected for sample collection was 3 years, with an equal distribution of children of age 1–5 years. Azithromycin MDA coverage in the study communities has been previously reported and exceeded 90% among children aged 6–59 months at each time point [18].

**Table 1:** Descriptive characteristics of communities (at baseline) and children providing dried blood spots for serologic assessments (at 36 months)

Study arm	Biannual azithromycin	Annual azithromycin
Community characteristics		
No. communities	12	12
No. children/community, mean	66 (range 36 to 124)	72 (range 37 to 119)
Proportion female, % (95% CI)	49.0% (45.1 to 52.9)	52.1% (49.3 to 54.8)
Age, months (95% CI)	18.7 (17.4 to 19.9)	18.4 (17.3 to 19.4)
Individual characteristics		
No. individuals	531	460
Age, years		
1	113 (21%)	99 (22%)
2	105 (20%)	87 (19%)
3	89 (17%)	84 (18%)
4	113 (21%)	86 (19%)
5	111 (21%)	104 (23%)
Female gender	275 (52%)	231 (50%)
*P. falciparum* infection	291 (55%)	256 (56%)
Clinically symptomatic *P. falciparum* infection plus fever	33 (6%)	36 (8%)
*P. falciparum* parasite density, median (IQR)	60 (0 to 1340)	100 (0 to 2960)
*P. falciparum* parasite density among children with infection, median (IQR)	1100 (240 to 5815)	2320 (470 to 8030)
Haemoglobin, g/dL, median (IQR)	9.5 (8.4 to 10.4)	9.4 (8.2 to 10.5)
MSP-1_19_ seroprevalence	333 (63%)	316 (69%)

**Fig. 2:**
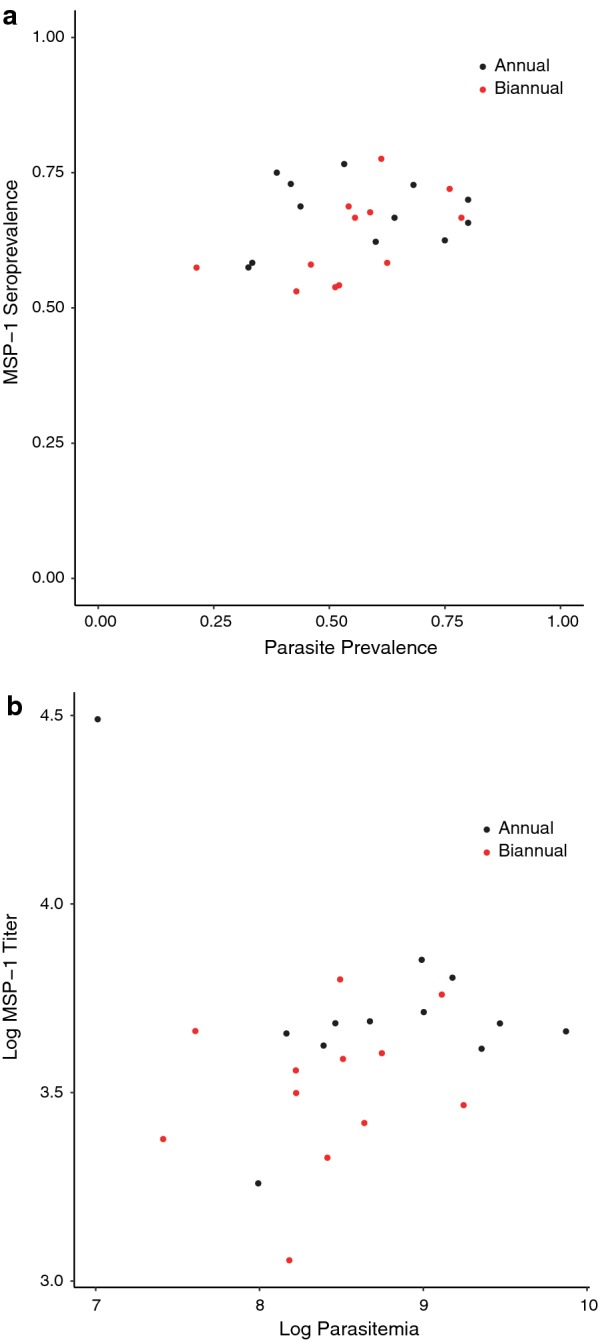
Correlation between community-level parasite prevalence and MSP-1_19_ seropositivity (**a**) and log-transformed parasite density versus log MSP-1_19_ antibody level (**b**). Community level measures are estimated from the proportion (**a**) or mean measurements (**b**) in 50 randomly-selected children aged 0–5 years per community. Red dots indicate biannually-treated communities, black dots indicate annually-treated communities

In September 2013 at the 36-month study visit, half of the children (55.2%, 95% CI 52.1 to 58.3%) had a positive thick smear for malaria parasites, and prevalence of malaria was 7.0% (95% CI 5.5 to 8.7%). As previously reported, there were no significant differences in malaria infection or clinically symptomatic malaria prevalence by study arm [18, 19]. Seropositivity against MSP-1_19_ was 65.5% (95% CI 62.5% to 68.4%). The prevalence of malaria infection and seropositivity increased with increasing age (PR 1.09 per one-year increase in age, 95% CI 1.06 to 1.13, *P *< 0.001), but the prevalence of clinically symptomatic malaria did not (PR 1.14 per 1-year increase in age, 95% CI 0.95 to 1.36, *P *= 0.16). Figure 2 shows the relationship between malaria seroprevalence and infection and parasite density and mean MSP-1_19_ titre. MSP-1_19_ seropositivity was higher among children with malaria infection (PR 1.23, 95% CI 1.04 to 1.44, *P *= 0.01) compared to those without infection, but not among those with clinically symptomatic malaria (PR = 1.20, 95% CI 0.70 to 1.59, *P *= 0.19) compared to those without clinically symptomatic malaria.

**Fig. 3:**
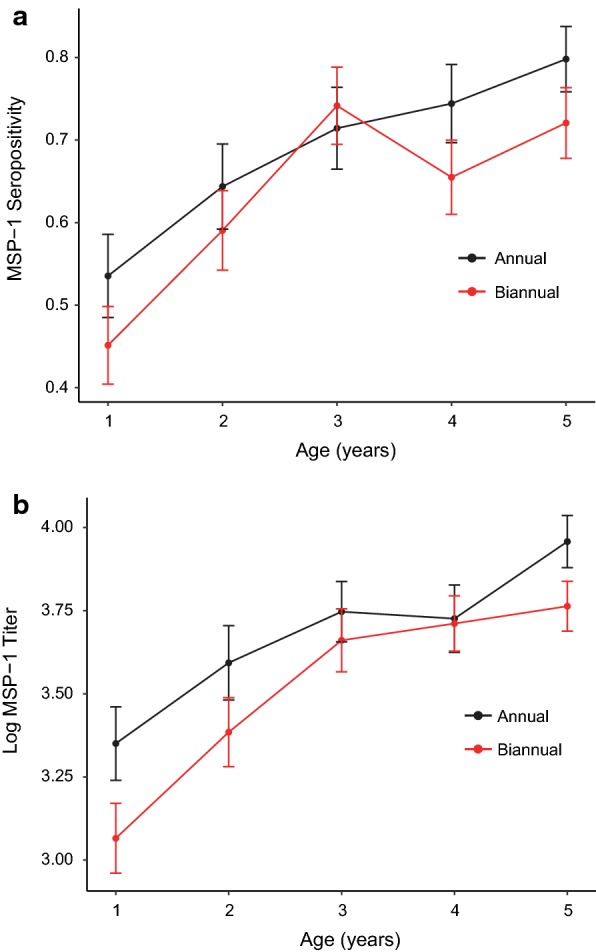
Age-seroprevalence curve (**a**) and age-quantitative antibody level curve (**b**) for biannually and annually-treated communities. Red lines indicate biannual communities, black lines indicate annual communities

MSP-1_19_ seroprevalence was 9% lower (PR 0.91, 95% CI 0.83 to 1.00, *P *= 0.047) among 1–5-year-olds from communities in which children received biannual treatment compared to those from communities receiving annual treatment. Mean MFI-BG was also lower in 1–5-year-olds in biannual MDA communities than children in communities receiving annual MDA (mean difference − 0.17, 95% CI − 0.02 to − 0.32, *P *= 0.04) (Fig. 3). There was no evidence that malaria exposure differed by age as assessed by age-seroprevalence (*P *= 0.94, age-by-arm interaction; Fig. 3) or MFI-BG by age curve (*P *= 0.35, age-by-arm interaction) between children in communities receiving biannual versus annual azithromycin.

## Discussion

Biannual distribution of azithromycin targeted to children was associated with lower antibody-based measures of exposure to *P. falciparum* compared to annual distribution to the entire community. In these same communities, no difference in *P. falciparum* infection prevalence after 36 months of treatment was reported [18]. The antibody response to MSP-1_19_ is long-lived [22] and is potentially a more sensitive indicator than microscopy point prevalence of cumulative malaria incidence. Thus, these results suggest that serologic markers of malaria exposure were slightly lower in areas receiving biannual compared to annual treatment with azithromycin.

The small (9%) reduction in MSP-1_19_ seroprevalence documented in this study between biannually and annually treated communities diverges from previous reports that have indicated a 44 to 73% decrease in malaria infection prevalence 1 month after azithromycin treatment [11, 34]. Compared with short-term effects of azithromycin on malaria infection, more modest reductions in antibody-based measures of *P. falciparum* exposure would be expected since the outcomes reflect differences in cumulative exposure between groups, averaged over a longer period of time. However, the intensity of transmission in holoendemic regions may make it difficult to detect differences in malaria seropositivity, particularly as children age and their probability of previous exposure increases. In holoendemic settings, short-acting interventions may also have less effect on transmission due to the ubiquity of infection. Among young children in particular, antibody responses may be lost, which could affect sensitivity to seasonal trends [35, 36]. The results of this study suggest that longer-term effects of azithromycin for malaria may be more modest than short-term effects.

Azithromycin has modest in vitro activity against *P. falciparum* due to targeting the plasmodial apicoplast [15, 16, 37], and thus a reduction in malaria burden is a plausible mechanism for the reduction in mortality observed following azithromycin distribution [1, 12, 13, 38]. In the same study area as the present analysis, biannual mass azithromycin distribution led to a reduction in mortality due to infection causes among preschool children. No difference in mortality due to malaria was noted, but this assessment was based on verbal autopsy [38], which can suffer from misclassification and poor sensitivity for malaria diagnosis [39, 40]. Additionally, the study was not powered to detect a difference in malaria-specific mortality. The results shown here suggest that malaria incidence is impacted by additional doses of azithromycin, which may indicate that reduction in malaria contributes to observed decreases in all-cause mortality following azithromycin distribution. However, the precise mechanism of action for the observed reduction in mortality following azithromycin distribution remains unclear. Some evidence suggests that the effect of azithromycin on malaria prevalence is transient [41]. Azithromycin likely also leads to reductions in other pathogenic organisms which may contribute to overall mortality effects. For example, the prevalence of *Campylobacter* species, an important cause of diarrhea and diarrhea-related mortality, was reduced in children in communities receiving biannual azithromycin compared to placebo in Niger [42]. Any effect of azithromycin on mortality that is mediated via changes in malaria transmission likely occurs in the context of alteration of transmission of other pathogenic organisms, and the overall effect of azithromycin distribution on mortality may or may not be due in part to small changes in malaria transmission.

The results of this study must be considered in the context of several limitations. First, there was no untreated control group, and thus the quantitative impact of azithromycin distribution cannot be determined. Second, biannual treatment of children under age 12 was compared to annual treatment of all individuals in the community, and so did not compare biannual versus annual treatment in the same age group. Third, mass azithromycin distributions occurred at the beginning of the high transmission season and the low transmission season. Malaria transmission is highly seasonal in Niger, and treatment targeted specifically during the high transmission season may have had a larger effect than treatment during the low transmission season. However, a single dose of azithromycin during the high transmission season may also paradoxically have reduced effect compared to treatment during the low transmission season due to the high parasite load and frequency of infection during the high transmission season [43]. Fourth, the generalizability of these results is limited only to preschool children in the setting of highly seasonal malaria transmission. Future studies could evaluate the impact of azithromycin MDA on malaria incidence in older children or adults.

## Conclusions

The results of this study are consistent with a small reduction in *P. falciparum* exposure in communities in Niger receiving targeted biannual azithromycin in children compared to those receiving annual azithromycin distribution to entire communities. Thus, mass treatment with azithromycin may lead to a decrease in malaria incidence in young children, presumably contributing to the survival benefits afforded by azithromycin.

## Data Availability

The datasets used during the current study are available from the corresponding author on reasonable request.
